# Ectopic expression of testis-specific transcription elongation factor in driving cancer

**DOI:** 10.1126/sciadv.ads4200

**Published:** 2025-03-14

**Authors:** Bin Zheng, Marta Iwanaszko, Shimaa Hassan AbdelAziz Soliman, Yukitomo Ishi, Sarah Gold, Ruxuan Qiu, Benjamin Charles Howard, Madhurima Das, Zibo Zhao, Rintaro Hashizume, Lu Wang, Ali Shilatifard

**Affiliations:** ^1^ Simpson Querrey Institute for Epigenetics and the Department of Biochemistry and Molecular Genetics, Northwestern University Feinberg School of Medicine, Chicago, IL 60611, USA.; ^2^ Department of Pediatrics, Northwestern University Feinberg School of Medicine, Chicago, IL 60611, USA.; ^3^ Department of Pediatrics, University of Alabama at Birmingham, Birmingham, AL 35233, USA.

## Abstract

The testis-specific BET protein BRDT structurally resembles the ubiquitous BRD4 and is misexpressed in cancer, and we show that BRDT misexpression may affect lung cancer progression. BRDT knockdown in lung cancer cells slowed tumor growth and prolonged survival in a xenograft model. Comparative characterization of PTEFb complex participation and chromatin binding indicates BRD4-redundant and BRD4-distinct BRDT functions. Unlike dual depletion, individual BRD4 or BRDT knockdown did not impair transcriptional responses to hypoxia in BRDT-expressing cells, consistent with redundant function. However, BRD4 depletion/BRDT complementation revealed that BRDT can also release paused RNA polymerase II independently of its bromodomains as we previously demonstrated not to be required for Pol II pause/release function of BRD4, underscoring the functional importance of the C-terminal domains in both BRD4 and BRDT and their potential as therapeutic targets in solid tumors. Based on this study, future investigations should explore BRD4-distinct BRDT functions and BRDT misexpression driving cancer pathogenesis.

## Teaser

BRDT, a testis-specific BET protein, is ectopically expressed in lung cancer, promoting hypoxia response and tumor progression.

## INTRODUCTION

Transcriptional regulation is essential for appropriate cellular responses to variable growth conditions and other external stimuli. In metazoans, which require precise spatiotemporal regulation of transcription for proper development, the physical release of RNA polymerase II (Pol II) from a promoter-proximal pausing elongation control “checkpoint” is a key regulatory step for the transcription of thousands of genes (*1*, *2*). Misregulation of this pause-release step, which occurs due to mutations that interfere with or alter the function of elongation factors, may drive or contribute to the pathogenesis of human cancers (*3*). One prominent example is seen in mixed lineage leukemia (MLL), in which 11q23 translocation-driven oncofusions of the gene *histone-lysine *N*-methyltransferase 2A* (*KMT2A*) frequently occur with genes encoding components of the cyclin-dependent kinase 9 (CDK9)–containing super elongation complex (SEC) to create fusion proteins (e.g., MLL-AF9) that contribute to leukemogenesis by misdirecting SEC’s Pol II–releasing activity, hyperactivating transcription at MLL-controlled loci (*4*–*6*).

The CDK9-containing elongation factor complex bromodomain-containing protein 4 (BRD4)–PTEFb (formed by BRD4 and the positive transcription elongation factor) is a master transcriptional regulator required for release of promoter-proximal paused Pol II into elongation at the majority of genes under normal conditions in somatic cells (*7*–*12*). However, recent studies have also demonstrated an essential role for the testis-specific BRD4 paralog bromodomain testis-specific protein (BRDT; in concert with the transcription factor A-MYB) in Pol II pausing and elongation control during meiosis in spermatogenic germ cells (*13*–*15*). BRDT was first identified as a cancer-testis antigen in lung cancers and however was later found to be ectopically expressed in other various cancer types (*16*, *17*). Ectopically expressed BRDT has been implicated as a transcriptional regulator for a set of genes driving cell proliferation in ovarian cancer and for a distinct set of genes driving cell migration in esophageal cancer, suggesting cancer context-specific roles (*18*, *19*). However, the functional role and impact of ectopic BRDT expression in lung cancer remain to be elucidated.

Similar to BRD4, BRDT is a member of the BET (bromodomain and extraterminal domain) family of transcription factors, which also includes BRD2 and BRD3. Each BET protein has two tandem bromodomains (BD), which bind to acetylated histone lysines with high affinity, and an extraterminal domain (ET), which is thought to mediate transcription factor recruitment. However, only BRD4 and BRDT have the C-terminal domain that, at least for BRD4, is known to be required for interaction with the PTEFb subunits CDK9 and cyclin T1 (CCNT1) (*20*, *21*). Despite the structural similarities between BRD4 and BRDT, it is unclear whether the functions of ectopically expressed BRDT are unique and/or redundant to the functions of the ubiquitously expressed BRD4 in cancer cells.

Here, we use in vitro and in vivo models to identify ectopically expressed BRDT as a factor promoting tumor growth, indicating BRDT as a potential therapeutic target in lung cancers with ectopic BRDT expression. Mechanistically, we show that ectopically expressed BRDT forms an active PTEFb complex that colocalizes with active transcription in lung cancer cells; that it can compensate for BRD4 loss in the transcriptional response to hypoxic stress; and that, similar to BRD4, it can release paused Pol II independently of its bromodomains. Based on these findings, we propose that targeting the C-terminal PTEFb-interacting domain of BRDT could be a more effective therapeutic strategy than bromodomain targeting in cancers where BRDT is ectopically expressed.

## RESULTS

### BRDT is ectopically misexpressed in human lung cancers

**Fig. 1.**
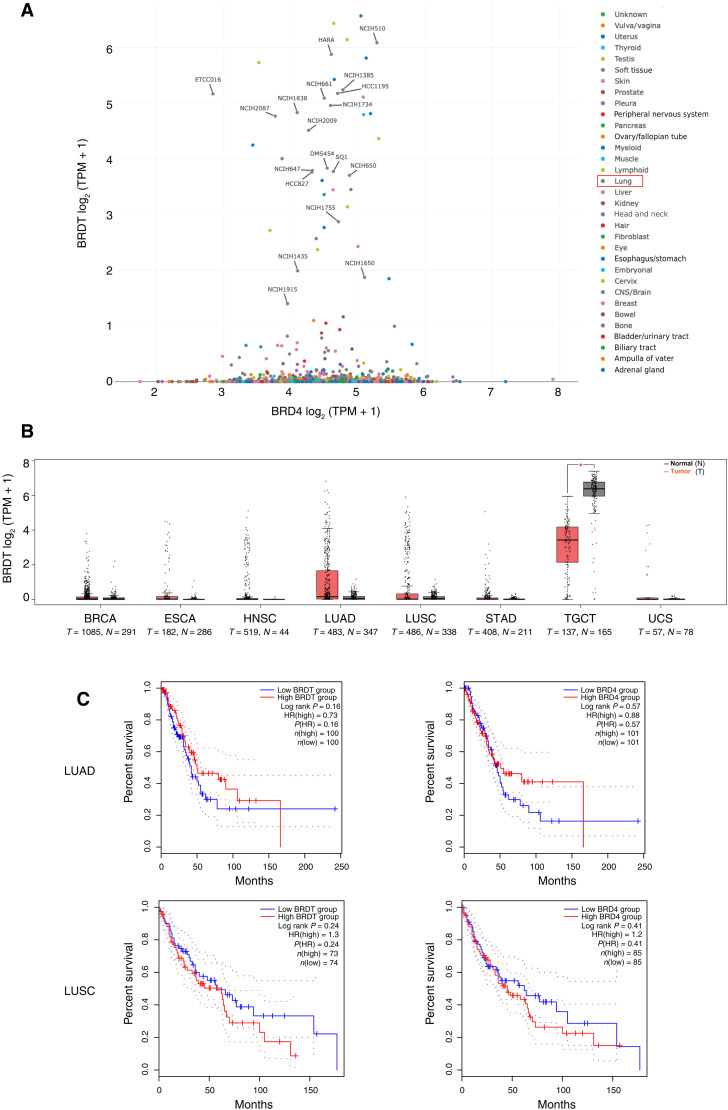
BRDT is ectopically expressed in lung cancers. (**A**) Plot showing the transcript expression levels of the ubiquitously expressed BRD4 versus the testis-specific BRDT across different cancer cell lines. Lung cancer–derived cell lines are labeled. Expression levels are presented as log_2_-transformed TPM (transcripts per million) with a pseudocount of 1 [log_2_ (TPM + 1)]. (Expression data downloaded from DepMap.). (**B**) Elevated gene expression of BRDT in various solid tumors (T, in red) compared to matched normal tissue samples (N, in black) from the TCGA project database (analyzed via the GEPIA2 web server). BRCA (breast-invasive carcinoma), ESCA (esophageal carcinoma), HNSC (head and neck squamous cell carcinoma), LUAD, LUSC, STAD (stomach adenocarcinoma), TGCT, and UCS (uterine carcinosarcoma). Matched normal samples were obtained from TCGA and Genotype-Tissue Expression Program (GTEx). Expression levels are presented as log_2_ (TPM + 1). (**C**) Kaplan-Meier curves comparing overall survival in patients with defined subtypes of LUAD (proximal inflammatory, proximal proliferative, and terminal respiratory unit) or LUSC (basal expression, classical expression, primitive expression, and secretory expression) whose tumors express low (below median, blue) versus high (above median, red) levels of BRDT (left) or BRD4 (right). The 95% confidence intervals are plotted as dashed lines. The *y* axis represents the percentage of patients surviving (percent survival) at a given time interval after diagnosis, represented on the *x* axis (months). HR was calculated on the basis of the Cox proportional hazards model, taking the high expression group as the intervention group [HR (high)].

Because BRDT has been presumed to function similarly to BRD4, we initially investigated a potential compensatory role for BRDT in cancer. We analyzed the expression level of BRDT compared to BRD4 across different cancer cell lines (*n* = 1479) and found no correlation between expression of BRDT and BRD4. However, we observed that more than 40% (*n* = 19) of the lines with robust BRDT expression (TPM ≥1, *n* = 45) were derived from samples of patients with lung cancer (Fig. 1A). To further explore the role of BRDT in lung cancer, we then analyzed BRDT expression in patient samples within The Cancer Genome Atlas (TCGA) database. BRDT expression levels were significantly decreased in testicular germ cell tumors (TGCTs) relative to matched normal tissue controls (Fig. 1B). However, BRDT is also ectopically expressed in several non-testis cancer types, most notably in lung adenocarcinoma (LUAD) and lung squamous cell carcinoma (LUSC), which are two major types of non–small cell lung cancer (Fig. 1B). Together, LUAD and LUSC account for more than 65% of all lung cancers (*22*).

Next, we used TCGA data to perform comparative analysis of overall survival for patients with high versus low BRDT expression. Across eight different cancer types including LUAD and LUSC, there was no clear association between BRDT expression level and hazard ratio (HR) except for testicular germ cell cancer (TGCT), where BRDT is endogenously expressed (fig. S1A). In TGCT, higher BRDT expression appeared to be associated with increased HR (≫1), whereas higher BRD4 expression appeared to be associated with decreased HR (≪1) (fig. S1A). The Kaplan-Meier analysis of overall survival in all patients with LUAD or LUSC (including both defined and undefined subtypes) revealed no clear impact of BRDT (or BRD4) expression on overall survival (fig. S1B). When limiting the analysis to patients with defined LUAD and LUSC subtypes, potential associations between BRDT expression and overall survival became apparent but were not statistically significant (Fig. 1C). However, the impacts of high BRDT expression on overall survival were notable and statistically significant (HR = 6.3, *P* = 0.0063; log rank *P* = 0.0022) for the secretory subtype of LUSC (fig. S1C). Together, these data led us to hypothesize that ectopic BRDT expression might contribute to tumor growth or progression in certain forms of lung cancer.

### Dox-induced BRDT knockdown impairs tumor growth in vitro and in vivo

**Fig. 2.**
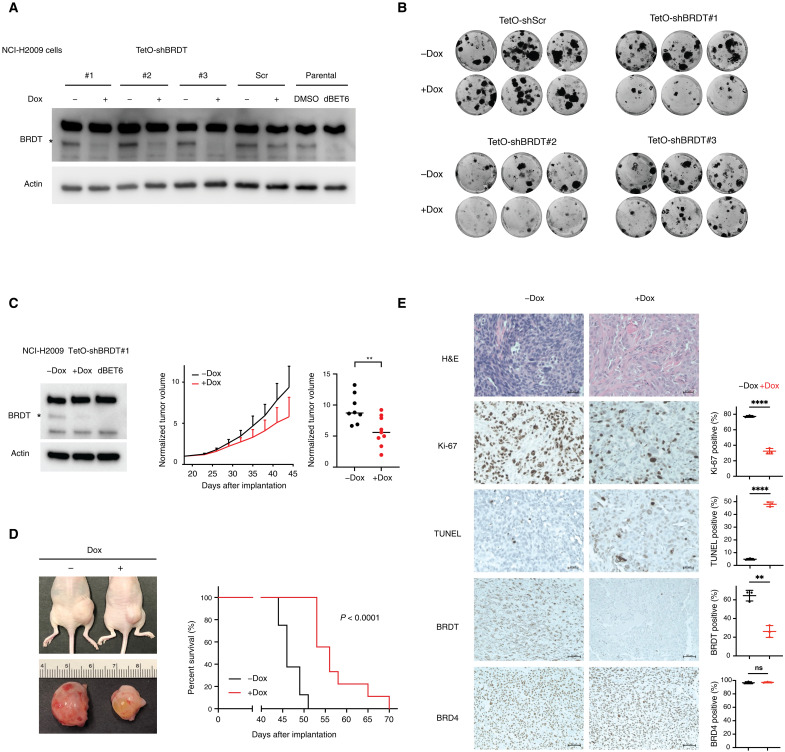
Dox-induced BRDT knockdown impairs tumor growth in vitro and in vivo. (**A**) Western blot analysis of Dox-induced BRDT knockdown in LUAD-derived NCI-H2009 cells with stable integration of BRDT-targeting (#1 to #3) or nontargeting (Scr) TetO-shRNA cassettes. dBET6 treatment in parental cells served as a positive control for BRDT depletion. (**B**) Colony formation assay in NCI-H2009 cells with integrated TetO-shRNA cassettes treated with or without Dox. (**C**) Left: Western blot confirming Dox-induced shRNA knockdown of BRDT expression in NCI-H2009 TetO-shBRDT cells before subcutaneous implantation into nude mice. Middle: Normalized tumor volume over the course of post-implantation treatment with phosphate-buffered saline (PBS) (*n* = 8, black) or Dox (*n* = 9, red). Right: Normalized tumor volume at day 44 post-implantation. BRDT knockdown significantly decreased NCI-H2009 xenograft tumor volume (**P = 0.0069, unpaired *t* test). (**D**) Left: Representative images of mice and excised tumors following treatment with PBS or Dox. Right: Kaplan-Meier curves comparing overall post-implantation survival. BRDT knockdown significantly increased overall survival in mice with NCI-H2009 xenograft tumors (****P < 0.0001, log rank test). (**E**) Representative histopathological staining of tumor tissue from mice treated with PBS (left) or Dox (right). Hematoxylin and eosin (H&E) staining is shown at top. IHC (with hematoxylin counterstain) is shown for the proliferation marker Ki-67, the apoptosis marker TUNEL, BRDT, and BRD4, with quantification of IHC-positive cells in four high-powered fields in each tumor (mean and SD shown). Scale bars, 50 μm (for BRDs) and 20 μm (others). Statistical analysis was performed using the unpaired *t* test. BRDT knockdown significantly reduced % Ki-67^+^ cells (****P < 0.0001) and significantly increased % TUNEL^+^ cells (****P < 0.0001). Significant reduction in % BRDT^+^ cells (**P = 0.0015) confirmed successful BRDT knockdown in vivo. No change was observed in % BRD4^+^ cells.

To evaluate our hypothesis regarding a potential role for BRDT in tumor growth or progression, we first generated a polyclonal antibody against BRDT (fig. S2A) and used it to confirm that ectopic BRDT expression is robustly detectable in cell lines derived from patients with small cell lung cancer, LUAD, and LUSC (fig. S2B). Next, to directly interrogate a role for BRDT in supporting tumor growth, we generated several cell lines with doxycycline (Dox)–inducible short hairpin RNAs (shRNAs) in the LUAD-derived NCI-H2009 background, which has been reported to readily generate xenograft tumors in nude mice (*23*). Although our homemade BRDT antibody has several nonspecific bands, using dBET6 treatment (which degrades BRDT) as a positive control confirmed specific knockdown of BRDT upon Dox treatment in these shBRDT lines (Fig. 2A). Following these validation steps, we tested the impact of BRDT knockdown on colony formation and observed a notable reduction in the number and size of colonies formed in the presence of Dox, whereas shCtrl had no effect, excluding possible confounding effects of Dox treatment (Fig. 2B). We then investigated the role of BRDT in tumor growth in vivo by picking a representative shBRDT line, reconfirming knockdown efficiency, and injecting cells subcutaneously into nude mice to generate xenograft tumors (Fig. 2C). Compared to the untreated control group, tumor growth was significantly impaired, and the overall survival was significantly extended in the Dox-treated BRDT knockdown group (Fig. 2, C and D). Last, we performed immunohistochemistry (IHC) on tissue slices from the excised xenograft tumors, first staining for BRD4 and BRDT to confirm specific knockdown of BRDT. Further IHC staining revealed lower levels of the proliferation marker Ki-67 and higher levels of the apoptosis marker TUNEL (terminal deoxynucleotidyl transferase–mediated deoxyuridine triphosphate nick end labeling) upon BRDT knockdown in these tumors (Fig. 2E). In addition to indicating that loss of ectopic BRDT expression due to shRNA knockdown in NCI-H2009 cells impairs xenograft-derived tumor growth, these data suggest proproliferative and/or anti-apoptotic functions for BRDT when it is ectopically expressed in cancer.

### BRDT and BRD4 form both similar and distinct complexes with PTEFb on active chromatin

**Fig. 3.**
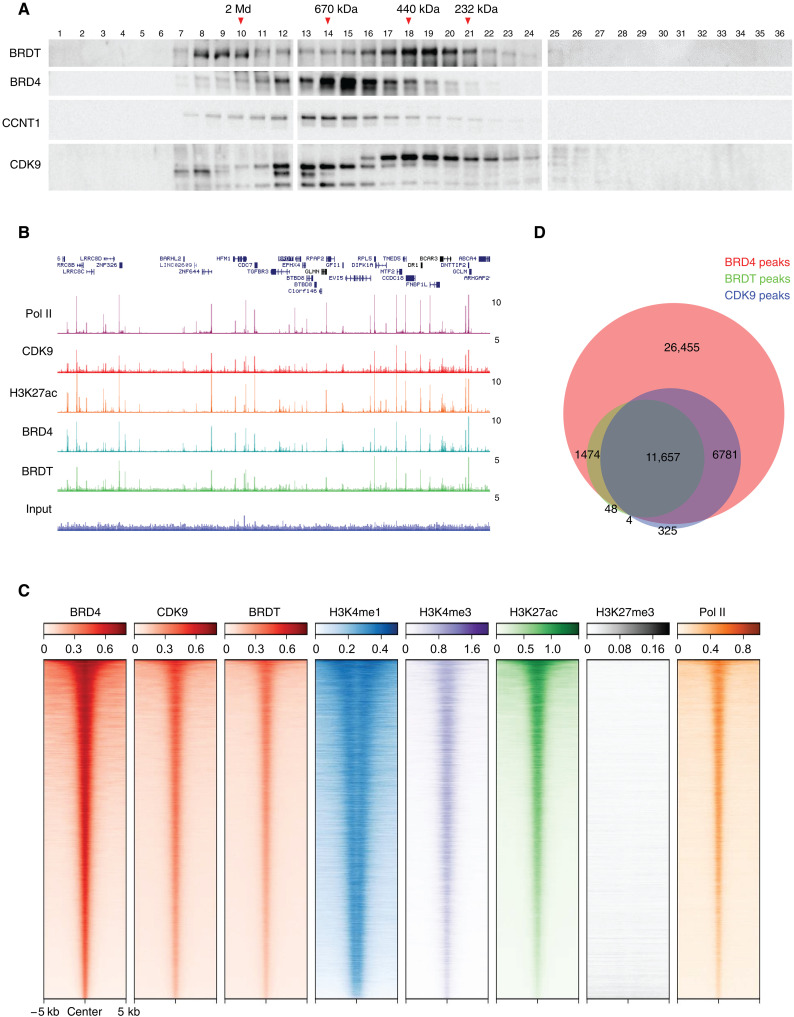
BRDT and BRD4 form similar and distinct complexes with PTEFb on active chromatin. (**A**) Western blot for BRDT, BRD4, CCNT1, and CDK9 after size exclusion chromatography performed using benzonase-treated whole nuclear extracts from NCI-H510 cells. (**B**) Representative track example of ChIP-seq signal in NCI-H510 cells visualized with zoom-out view, showing colocalization of BRDT, BRD4, Pol II, and CDK9 at active chromatin regions marked by H3K27ac. (**C**) Global heatmaps of ChIP-seq signals for BRD4, CDK9, BRDT, and Pol II indicating their genome-wide colocalization at active chromatin regions (marked by H3K4me1, H3K4me3, and H3K27ac and lack of H3K27me3). (**D**) Venn diagram for peak overlaps among BRD4, BRDT, and CDK9 genome wide.

BRDT shares structural similarities including a putative PTEFb-interacting C-terminal motif with its related BET family member BRD4, which is a master regulator of Pol II transcription (fig. S2A). Despite these structural similarities, the function of ectopically expressed BRDT and its impact on Pol II transcription remain unclear. To comparatively characterize BRDT-PTEFb and BRD4-PTEFb complexes, we first conducted size exclusion chromatography using whole nuclear extracts from NCI-H510 lung cancer patient-derived cells, which express relatively high levels of BRDT at both the mRNA and protein level (Fig. 1A and fig. S2B). We observed mutually exclusive interactions of BRDT and BRD4 with the PTEFb subunits CCNT1 and CDK9 in distinct complexes, suggesting that BRDT-PTEFb and BRD4-PTEFb may have distinct and independent functions (Fig. 3A). To further characterize BRDT-PTEFb versus BRD4-PTEFb function at the chromatin level, we then performed chromatin immunoprecipitation sequencing (ChIP-seq) in NCI-H510 cells. We observed widespread colocalization of BRDT, BRD4, and CDK9 at active chromatin regions (Fig. 3, B and C), with the vast majority of BRDT and CDK9 peaks overlapping BRD4 peaks (Fig. 3D). Pathway enrichment analysis (fig. S2C) using total peaks from BRD4 and BRDT ChIP-seq revealed both shared and distinct pathways regulated by BRD4 and/or BRDT. Genome feature distribution analysis (fig. S2D) revealed a bias toward promoter-proximal BRDT peaks relative to a broader distribution of BRD4 peaks among different genomic features, consistent with BRD4 function at enhancers. Together, these results suggest that ectopic BRDT expression in tumors might (i) confer BRDT-specific functions and (ii) compensate for or augment some redundant subset of BRD4 functions.

### BRDT can compensate for loss of BRD4 in the transcriptional response to hypoxic stress

**Fig. 4.**
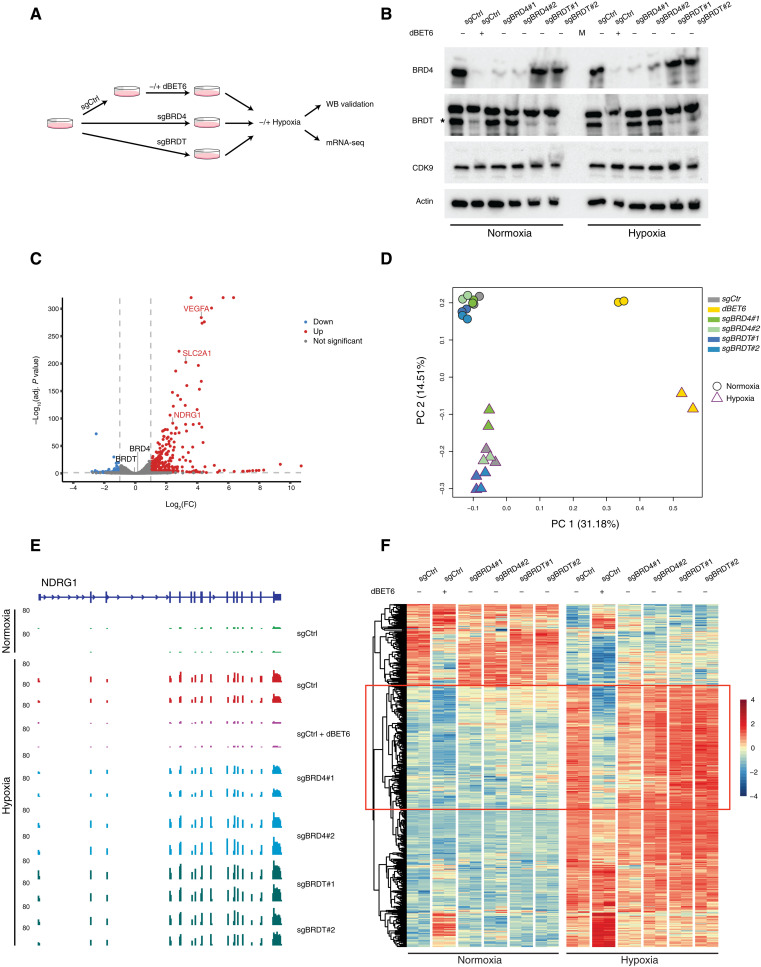
BRDT can compensate for loss of BRD4 in the transcriptional response to hypoxic stress. (**A**) Schematic diagram of the experimental design for evaluating the impact of BRD4, BRDT, or dual BRD4/BRDT loss on transcriptional responses to hypoxia. (**B**) Western blot for BRD4, BRDT, and CDK9 in NCI-H510 cells under conditions of normoxia (~18% oxygen, left) or hypoxia (1% oxygen, right) after transfection of sgRNA targeting BRD4 or BRDT, showing the efficiency of BRD4 or BRDT knockdown by each sgRNA. Nontargeting sgRNA (sgCtrl) is the negative control, and dBET6 treatment (dual depletion of both BRD4 and BRDT) is the positive control for knockdown efficiency at the protein level. (**C**) Volcano plot of genes differentially expressed in response to hypoxic stress in the cells described in (B), with representative hypoxia-induced genes highlighted. Neither BRD4 nor BRDT was affected by hypoxia. (**D**) PCA analysis of RNA-seq data in cells cultured under normoxic or hypoxic conditions after depletion of either BRD4 or BRDT via individual sgRNA or after dual depletion of both BRD4 and BRDT by dBET6 treatment [as described in (B)]. (**E**) Track visualization of RNA-seq signal at the representative hypoxia-responsive gene *NDRG1* locus showing transcriptional activity induced by hypoxia that was lost upon simultaneous depletion of BRD4 and BRDT by dBET6 treatment but remained upon individual depletion of either BRD4 or BRDT by sgRNA. (**F**) Heatmap showing a cluster of hypoxia-responsive genes for which transcriptional induction in response to hypoxia was strongly diminished by dBET6 treatment, but not by sgRNA targeting either BRD4 or BRDT. PC, principal component.

It has been well-demonstrated that BRD4 serves as a master regulator for global release of promoter-proximal paused Pol II and that it does so by recruiting PTEFb, which in turns phosphorylates the C-terminal domain of Pol II. Either BRD4 depletion or CDK9 inhibition leads to Pol II pausing at promoters, genome wide (*8*, *10*, *12*). We have also demonstrated that when cells encounter the heat shock stress condition, BRD4 is sidelined and SEC acts as the essential PTEFb-recruiting mediator of Pol II release at heat shock–responsive genes (*10*). However, this is not the case when cells encounter hypoxia (low oxygen tension): We recently identified BRD4 but not SEC as required for the gene-specific transcriptional response to hypoxic stress (*24*). Hypoxia is particularly common in the core of solid tumors (*25*, *26*). Therefore, to investigate potential functional redundancy between BRD4 and BRDT in BRDT-expressing cancer cells, we decided to assess the impact of BRD4 versus BRDT loss on the transcriptional response to hypoxic stress. First, we generated separate BRD4 and BRDT knockdown lines in the NCI-H510 background using CRISPR-Cas9 and two individual single-guide RNAs (sgRNAs) for either factor (Fig. 4A). We also used the BET-specific degrader dBET6 to rapidly deplete both BRD4 and BRDT in a subset of sgCtrl cells. After six hours of culture in normoxic (~18% O_2_) or hypoxic (1% O_2_) conditions, we harvested sgCtrl, sgCtrl + dBET6, sgBRD4, and sgBRDT cells and divided the cell pellet into two portions, one for Western blot to validate knockdown efficiency and another for RNA sequencing (RNA-seq) to assess the impact of knockdown of these two factors on the transcriptional response to hypoxic stress (Fig. 4A). Depletion of bands corresponding to BRD4 and BRDT indicated that knockdown via sgRNA was successful (Fig. 4B). We then performed RNA-seq, which confirmed successful knockdown (fig. S3A) and captured hypoxia-induced gene expression changes, including hundreds of genes up-regulated after 6 hours of hypoxia, as well as a smaller number of down-regulated genes (Fig. 4C). Principal components analysis (PCA) analysis of RNA-seq data confirmed that cells in conditions of hypoxia or normoxia were transcriptionally distinct and that cells treated with dBET6 (dual BRD4/BRDT depletion) were transcriptionally distinct from cells with individual knockdown of either BRD4 or BRDT (Fig. 4D). Gene Ontology (GO) analysis also confirmed that the top pathways enriched among the hypoxia-induced transcripts in untreated sgCtrl cells were all related to hypoxic response (fig. S3B). Clustering of the hypoxia-responsive genes in sgCtrl cells revealed a large subset of genes for which transcriptional induction failed upon dBET6 treatment but not upon individual knockdown of BRD4 or BRDT, indicating functional redundancy between these two factors in the transcriptional response to hypoxia (Fig. 4, E and F). Last, GO term analysis revealed that the top pathways enriched among this redundantly BRD4/BRDT-regulated subset were all related to angiogenesis (fig. S3C).

### BRDT releases promoter-proximal paused Pol II in a manner similar to that of BRD4

**Fig. 5.**
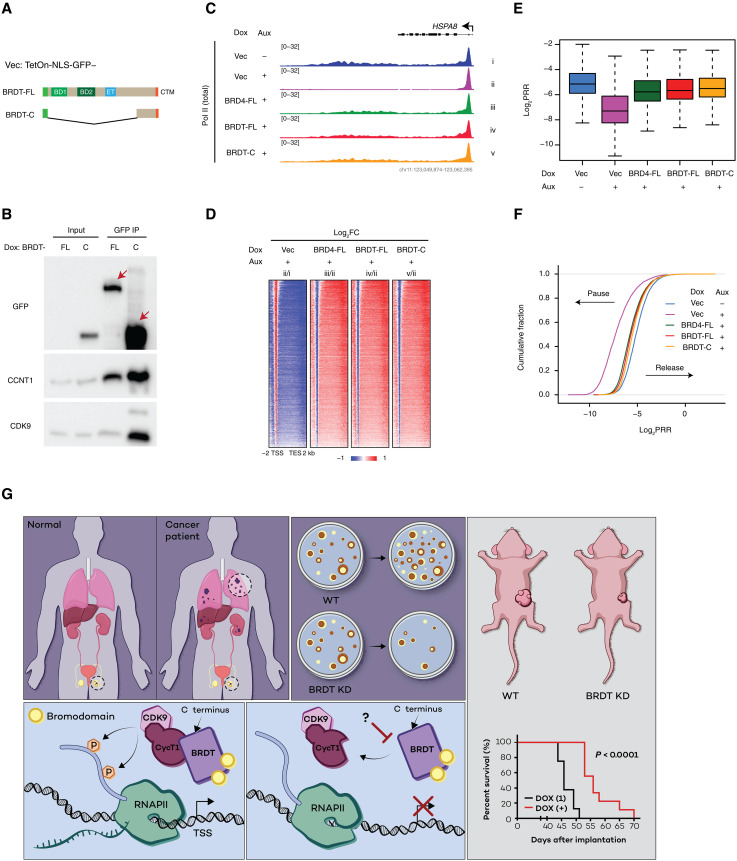
BRDT releases promoter-proximal paused Pol II in a manner similar to that of BRD4. (**A**) Schematic diagram of the Dox-inducible, N-terminally GFP-tagged BRDT constructs BRDT-FL (full-length BRDT) and BRDT-C (bromodomain-less C-terminal BRDT fragment). (**B**) Western blot for GFP and the PTEFb subunits CCNT1 and CDK9 after immunoprecipitation of the GFP-tagged BRDT constructs showing that both BRDT-FL and BRDT-C can interact with PTEFb. (**C**) Representative track example at the *HSPA8* gene locus of Pol II ChIP-seq showing Pol II pausing upon depletion of endogenous BRD4 by auxin treatment and comparable rescue by BRD4-FL, BRDT-FL, and BRDT-C. (**D**) Heatmap of log_2_FC in Pol II occupancy for the rescue experiment in (C) showing a genome-wide increase in Pol II signal at promoters and a decrease in signal at gene bodies upon auxin-induced BRD4 depletion. BRD4-FL, BRDT-FL, and BRDT-C all rescue the genome-wide shift in Pol II occupancy in a similar manner. (**E**) Boxplot of log_2_PRR calculated from Pol II ChIP-seq signal at promoters versus gene bodies for the rescue experiment in (C). (**F**) Empirical cumulative distribution function plot of log_2_PRR for the rescue experiment in (C). (**G**) Putative model for ectopically expressed BRDT in lung cancer promoting tumor progression. Testis-specific BRDT has also been found in multiple cancers, with especially high expression levels found in common forms of lung cancer. BRDT knockdown (KD) impairs colony formation for lung cancer cells in vitro and reduces xenograft tumor growth in vivo, extending animal survival. BRDT can mediate gene expression by partnering with PTEFb to release paused Pol II, similar to BRD4, and BRDT can also functionally compensate for BRD4 in the transcriptional response to hypoxia. The C terminus of BRDT could be a potential therapeutic target for solid tumors, where hypoxic gene expression is critical.

We have recently demonstrated that BRD4 mediates the release of promoter-proximal paused Pol II in a bromodomain-independent but C-terminal domain–dependent manner (*27*). In addition to sharing structural similarities with BRD4, including a PTEFb-interacting C-terminal domain that is absent in BRD2 and BRD3 (Fig. S2A), BRDT also shares a redundant function in the transcriptional response to hypoxia (Fig. 4 and fig. S3). We therefore hypothesized that BRDT might mediate the release of promoter-proximal paused Pol II in a manner similar to that of BRD4. To test this hypothesis, we first generated lentivirus-packaged, Dox-inducible, and green fluorescent protein (GFP)–tagged constructs of full-length BRDT and a bromodomain-less C-terminal BRDT fragment (Fig. 5A). The C-terminal BRDT fragment was the same length as the C-terminal BRD4 fragment that we previously demonstrated to be sufficient to release Pol II (*27*). Next, we used our BRD4-IAA7 degron system in the BRDT-null DLD-1 line, which enables rapid BRD4 depletion and Pol II pausing upon auxin treatment (*27*). After stably integrating the GFP-tagged BRDT constructs in the BRD4-IAA7 DLD-1 background, we induced their expression and carried out GFP immunoprecipitation (IP) to confirm that BRDT interacts with PTEFb and that this interaction is retained in the absence of BRDT bromodomains (Fig. 5B). After this validation step, we carried out a rescue experiment using our full-length BRD4 construct (*27*) as a control for rescue of pause-release (as assessed by Pol II ChIP-seq) after the depletion of endogenous BRD4 (Fig. 5, C to F). We confirmed the expression of the GFP-tagged BRD4 and BRDT constructs by Western blot using BRD4- or BRDT-specific antibodies (fig. S4A). Genomic integration and Dox-induced expression of the BRD4 and BRDT constructs were also confirmed by Pol II ChIP-seq reads that mapped to exons at the respective gene loci (fig. S4B). In the rescue experiment, BRD4 depletion caused accumulation of promoter-proximal paused Pol II (Fig. 5C), reflected in genome-wide Pol II pausing (Fig. 5D) that can be quantified as a pause-release ratio (log_2_PRR) of Pol II signal at gene bodies relative to promoters (Fig. 5, E and F). Pol II pausing was rescued as expected by the expression of the full-length BRD4 construct, and it was similarly rescued by the expression of either the full-length or the bromodomain-less C-terminal fragment BRDT constructs (Fig. 5, C to F). Together, these findings indicate that the bromodomain-independent function of BRD4 in the release of promoter-proximal paused Pol II is conserved in BRDT.

## DISCUSSION

In this study, we have investigated the impact and function of the testis-specific transcription factor BRDT when it is ectopically expressed in lung cancer. We focused on ectopic expression of BRDT in lung cancer, the context in which BRDT was first identified as a cancer-testis antigen, based on the trends we identified in our analysis of publicly available expression data (Fig. 1 and fig. S1). We observed consistently higher BRDT expression in cell lines derived from lung tumors relative to other lineages and highest levels of ectopic BDRT expression in lung tumors relative to other tumor types, but it is not clear whether BRDT expression in patient samples is entirely attributable to cancer cells or whether it might also reflect expression in tumor-associated immune cells. It is interesting to note that high expression of BRDT is associated with an increased HR in TGCTs, where BRDT expression is significantly lower than in normal tissue controls. However, while the lung cancer types LUAD and LUSC both ectopically express BRDT, high BRDT expression appears to have opposite impacts on HR in LUAD versus LUSC. Shedding some light on this apparently differential impact of ectopic BRDT expression, further analysis of BRDT isoforms revealed distinct patterns of isoform representation in these lung cancer types, including alternative full-length BRDT isoforms predicted to have an altered or absent first bromodomain primarily expressed in LUSC but not LUAD (fig. S4, D and E). Future studies will investigate the potential impacts and functions of BRDT isoforms in various forms of lung and other BRDT-expressing cancers.

We have demonstrated that loss of ectopic BRDT expression via Dox-inducible shRNA knockdown in lung cancer cells inhibited colony formation and growth in vitro, an effect that was recapitulated in vivo by decreased tumor size and prolonged survival in a xenograft model (Fig. 2). Although the impacts of BRDT loss on tumor size and survival were statistically significant, they were not particularly notable or complete. Given the apparent redundancy in BDRT and BRD4 function evidenced by our ChIP-seq studies (Fig. 3), the loss of ectopically expressed BRDT may be compensated for by BRD4. Alternatively, shRNA knockdown may not affect all BRDT isoforms.

Based on the structural similarity between BRDT and BRD4 and after demonstrating BRDT interaction with PTEFb and similar binding for BRDT and BRD4 genome wide (Fig. 3), we have examined the long-proposed yet untested redundancy between these bromodomain-containing factors. We did so by focusing on the transcriptional response to the solid tumor-relevant stress condition of hypoxia, in which we recently demonstrated that BRD4 plays a major role. In addition to demonstrating that BRDT can compensate for the loss of BRD4 in the transcriptional response to hypoxia (Fig. 4), we have also demonstrated that BRDT can mediate the release of promoter-proximal paused Pol II independently of its tandem bromodomains (Fig. 5), similar to the distinct “layer” of BRD4-PTEFb we recently identified as actively promoting release of paused Pol II without binding to histone lysine acetylation (*27*). Based on lessons learned from BET inhibitors (*28*–*31*), we would predict limited antitumor efficacy for inhibitors that target the BET bromodomains of ectopically expressed BRDT. Instead, the C-terminal region of BRDT should be considered as a therapeutic target if the goal is to interfere with BRDT-mediated release of paused Pol II.

Our findings do not indicate that Pol II release is either the sole or primary function of BRDT when it is ectopically expressed in cancer cells. Functional redundancy between BRDT and BRD4 is also context and gene specific. For example, our data indicate that expression of the transcription factor Myc is controlled more by BRD4 than BRDT (fig. S4C). Moreover, although BRD4 is also expressed in germ cells, it cannot compensate for loss of BRDT as a meiotic factor during spermatogenesis; both JQ1 treatment and BRDT depletion impair spermatogenesis, indicating that unlike its function as a Pol II–releasing factor, BRDT’s function as a meiotic factor is likely to be bromodomain dependent (*21*, *32*, *33*). The disparate nature of the bromodomains in BRDT and BRD4 could be an important difference contributing to the distinct, nonredundant functions of these BET proteins. Studies have shown that unlike BRD4, in which both bromodomains recognize acetylated histone tails, in BRDT, only the first bromodomain (BD1) binds with high affinity to histone lysine acetylation, while the second bromodomain (BD2) contributes a nonspecific interaction with DNA that is essential for BRDT function in meiotic and post-meiotic chromatin remodeling and genome reorganization (*21*, *34*). Further studies are required to determine the functions of BRDT bromodomains when BRDT is ectopically expressed in somatic cells. Bromodomains are altered or absent in some of the BRDT isoforms ectopically expressed in cancer (fig. S4, D and E). Short BRD4 isoforms (lacking the C terminus PTEFb-interacting domain but retaining the bromodomains and ET domain) have been well studied, with functions demonstrated to be different from or opposite to that of the full-length BRD4 isoform (*35*–*39*). Although BRDT and BRD4 expression levels are uncorrelated in lung cancer cell lines, the potential relationship between expression of full-length versus short BRD4 and BRDT isoforms is unclear. Distinct, nonredundant functions of BRDT and BRD4 could also be conferred by the formation of distinct complexes with PTEFb, as implicated by our size exclusion study (Fig. 3). We are currently working to elucidate the components and the functional significance of the two distinct BRDT-PTEFb complexes we have identified in BRDT-expressing lung cancer cells.

Last, it will be interesting to investigate the epigenetic mechanisms by which BRDT, normally restricted to the testis, comes to be ectopically expressed in neoplasia arising from somatic cells. We know that BRDT expression gradually increases during spermatogenesis, with detectable mRNA appearing at the early spermatocyte stage, but upstream regulators are unknown (*21*). Do the same mechanisms functioning in spermatogenesis turn on BRDT expression in lung and other BRDT-expressing cancers? To identify upstream regulators of BRDT, activation would not only elucidate the molecular mechanisms by which testis-specific transcription factors such as BRDT are ectopically expressed in cancer but might also reveal mechanisms of dysfunction in spermatogenesis. CRISPR screening is a reasonable route to achieve this goal.

## MATERIALS AND METHODS

### Cell culture

NCI-H510 (HTB-184), NCI-H1385 (CRL-5867), NCI-H838 (CRL-5844), NCI-H2009 (CRL-5911), and DLD-1 (CCL-221) cells were purchased from American Type Culture Collection. DLD-1 cells were cultured in Dulbecco’s modified Eagle’s medium (Corning, #10013CV), while other cell lines were in RPMI (Thermo Fisher Scientific, #A1049101). Both media were supplemented with 10% fetal bovine serum (Sigma-Aldrich, #F2442), 1% Penicillin-Streptomycin (Gibco, #15140122), and 1% GlutMAX (Gibco, #35050061). Cells were cultured in an incubator at 37°C with a supply of 5% CO_2_. Hypoxia induction was carried out in a Whitley H35 Hypoxystation with 1% O_2_.

### Western blot

Following procedures previously described (*27*), for whole-cell lysate Western, cells were directly lysed in 2X Laemmli sample buffer (Bio-Rad, #1610747) and boiled for 10 min before loading to the precast protein gels (Bio-Rad, #4561096). For GFP IP, cells were trypsinized and washed with phosphate-buffered saline (PBS) twice. Cell pellets were incubated with cold Triton X-100 lysis buffer [50 mM tris HCl (pH 8.0), 150 mM NaCl, 1.5 mM MgCl_2_, 10% glycerol, 0.5% Triton X-100, dithiothreitol, benzonase, protease inhibitor, and phosphatase inhibitor] and rotated at 4°C for ≥1 hour. EDTA (final concentration of 1.5 mM) was added to the cell lysate before centrifugation for 20 min at max speed at 4°C. Supernatant was collected and incubated with GFP-Trap magnetic beads (Proteintech, #gtma) at 4°C with rotation for ≥4 hours. After immunoprecipitation, beads were washed with cold Triton X-100 lysis buffer five times at 4°C and eluted with 2.5 M glycine buffer (pH 2.0) at room temperature for 5 min. A 1 M tris base pH > 10 was added to neutralize the eluate before equal volume of 4X Laemmli sample buffer was added. Samples were then boiled for 5 min for Western blot. BRD4 (#13440), BRD2 (#5848), β-actin (#3700), CDK9 (#2316), BRD3 (#94032), and cyclin T1 (#81464) antibodies were purchased from Cell Signaling Technology. GFP antibody (#sc-9996) was purchased from Santa Cruz Biotechnology. BRDT antibody was generated in house.

### Knockdown by shRNAs or sgRNAs

TetO-shRNA constructs were cloned using Age I and Eco RI sites in the Tet-pLKO-puro vector (#21915) obtained from Addgene. shRNA target sequences are as follows: TetO-shScr (CCTAAGGTTAAGTCGCCCTCGCTCGA), TetO-shBRDT#1 (CCGATGGATCTTGGAACTATT), TetO-shBRDT#2 (CCCTAAGTTTACAGAAGTAAA), and TetO-shBRDT#3 (CATCAGAAGCTCAAGATAAAT).

sgRNA constructs were cloned using Bsm BI v2 sites in the lentiCRISPR v2 vector (#52961) obtained from Addgene. The sgRNA target sequence information was obtained from the Addgene Brunello library. Target sequences are as follows: sgCtrl (GCACTACCAGAGCTAACTCA), sgBRD4#1 (CACCAAACTCCTGAGCATCA), sgBRD4#2 (CCAGACCCCTGTCATGACAG), sgBRDT#1 (AACTCCCTGGAGATAAACTT), and sgBRDT#2 (CAACTCCAGTTCACAAACTG).

Like previously described (*27*), all target sequences in the constructs were verified by Sanger sequencing (ACGT, Inc.) before lentiviral packaging in 293T cells with pspax2/pmd2.g plasmids. Transfection was carried out using the Lipofectamine 3000 Transfection Reagent (Invitrogen, #L3000001) in a six-well plate. Lentivirus was collected and filtered before infection. Infected cells were selected using Puromycin (Gibco, #A1113803) until no further cell death observed. To induce the shRNAs, 500 nM Dox (STEMCELL Technologies, #72742) was added to the cell culture and replaced every 2 days.

### BRD4 degron cell line and BRD4/BRDT mutant expressing lines

BRD4-IAA7 degron cells expressing Dox-inducible GFP and GFP-BRD4-FL were generated in previous study (*27*). Similarly, constructs for expressing Dox-inducible GFP-BRDT-FL and GFP-BRDT-C were cloned by using NEBuilder HiFi DNA assembly reaction [New England Biolabs (NEB), #E2621S] with synthetic gBlocks (Integrated DNA Technologies and Twist Biosciences). Lentivirus was packaged to infect the BRD4-IAA7 cells, and blasticidin (Gibco, #A1113903) was used to select transduced cells for 2 weeks. Mutant expression was achieved by adding 50 nM Dox to the cell culture for 2 days, and endogenous BRD4 depletion was achieved by treating the cells with Auxin (Abcam, #ab146403) for 3 hours.

### Colony formation assay

A total of 400 cells were seeded in each of the six-well plate. Dox treatment was started the next day and replaced together with fresh media every 2 days for 18 days. A 4% paraformaldehyde solution (Thermo Fisher Scientific, #50-980-495) in PBS was used to fix the cells at room temperature under shaking for 20 min. Plates were then washed three times gently with tap water and dried overnight, followed by violet solution (MilliporeSigma, #HT90132) staining for 20 min.

### Xenograft studies

Six-week-old female athymic mice (nu/nu genotype, BALB/c background) were purchased from Envigo and housed under aseptic conditions. NCI-H2009 TetO-shBRDT#1 cells were implanted into the flank of athymic mice as previously described (*40*). Briefly, 4 × 10^6^ cells, in 0.4 ml of cell culture media with Matrigel (BD Bioscience) at 1:1 ratio, were injected in the right flank of mice under anesthetization by inhaled isoflurane. Mice were randomly assigned to vehicle (no Dox, *n* = 8) and Dox treatment [20 mg/kg by intraperitoneal injection for 14 days followed by dox water (2 mg/ml) for 14 days, *n* = 9] groups when the size of tumor reached at 100 mm^3^ (18 days after implantation). The tumor sizes were measured on alternate days with calipers, and the mice were euthanized when the tumor size reached 1000 mm^3^. Dox used in the animal studies was purchased from Sigma-Aldrich (catalog no. D9891-25G). Subcutaneous tumors were collected from the mouse and fixed with 4% paraformaldehyde (PFA). PFA-fixed tumors were paraffin-embedded and sectioned (4 μm) for hematoxylin and eosin staining. For IHC staining, anti-Ki67 (2 μg/ml) (Ventana, Tucson, AZ, USA), BRD4 (1:1000; #13440, Cell Signaling Technology), and the home-made BRDT (1:1000; rabbit) antibodies were used. To assay the apoptotic response to treatment, TUNEL staining was performed using the DeadEnd Colorimetric TUNEL system (Promega, Madison, WI, USA) according to the manufacturer’s protocol. All animal protocols were approved by the Northwestern University Institutional Animal Care and Use Committee (#IS00000787).

### Chromatin immunoprecipitation sequencing

ChIP-seq experiments for DLD-1 cells were carried out as previously described (*27*). Briefly, cells in a 15-cm plate were crosslinked by 15 ml of 1% PFA (Thermo Fisher Scientific, #28908) in PBS with shaking for 10 min at room temperature. A 2 ml of 2.5 M gylcine was added to quench the reaction with continue shaking for 5 min. Crosslinked cells were then scraped and collected. Covaris E200 was used for chromatin sonication with following settings: 10% duty factor, 200 cycles per burst, and 140 peak intensity power for 10 min. For NCI-H510 cells, 5 μl of Rpb1 (Cell Signaling Technology, #14958), CDK9 (Cell Signaling Technology, #2316), H3K27ac (Cell Signaling Technology, #8173), BRD4 (Bethyl Laboratories, #A700-004), or 40 μl of BRDT anti-sera were incubated with the lysate overnight at 4°C with rotation for immunoprecipitation. Protein G–coupled Dynabeads (Invitrogen, #10004D) were used to pull down the immune complexes. Protease K (Roche, #3115828001) was used during reverse crosslink (65°C, 1200 rpm, overnight). DNA was then purified through QIAquick PCR Purification Kit (QIAGEN, #28106). Libraries were prepared by using the KAPA HTP library preparation kit (Roche, #07961901001) and sequenced on the Illumina NovaSeq 6000.

### RNA sequencing

NCI-H510 cells were cultured in rectangular flask (Corning, #430639). To deplete both BRD4 and BRDT, 250 nM dBET6 was added to the sgCtrl cells 3 hours before hypoxia induction. To carry out hypoxia induction, normoxia plates were kept in normal incubator, while hypoxia plates were moved to the hypoxia station. Six hours later, all plates were transferred to the cold room, and cell pellets were collected. The cell pellets (20%) were saved for Western blot and 80% for RNA extraction. Total RNAs were extracted using the RNeasy Mini Kit from QIAGEN (QIAGEN, #74106) according to the procedure. mRNAs were enriched using the NEBNext poly(A) mRNA magnetic isolation module (NEB, #E7490L). Libraries were prepared using the NEBNext Ultra II Directional RNA library preparation kit (NEB, #E7760L) and sequenced on the NovaSeq 6000 (*27*).

### Data analysis

The survival analysis based on the expression of BRDT and BRD4 genes was performed using data from TCGA implemented in GEPIA2 portal (*41*). BRDT isoforms were annotated using genome assembly GRCh38.p13 and Ensembl database, release 98 (*42*). Survival was calculated as the overall survival in months since the time of diagnosis, and the median gene expression was used for the high/low expression group cutoff and log rank test for hypothesis testing. HR calculation was based on Cox PH model with the 95% confidence interval. GEPIA2 was used to generate Kaplan-Meier plots.

#### Chromatin immunoprecipitation sequencing

ChIP-seq reads in the NCI-H510 cells were at least 30 M per sample and were aligned to hg19.

ChIP-seq reads in DLD-1 were aligned to hg38 with bowtie ½ (*43*). Following procedures previously described (*27*), A total of 6481 Pol II–transcribed genes with transcription start site (TSS) and transcription end site (TES) annotated were obtained from our previously published study (*44*). Heatmap for the log_2_ fold change (log_2_FC) of Pol II occupancy was generated using the deeptools with nondefault options: binSize of 10 and pseudocount of 0.1. PRR was calculated as the ratio of Pol II reads density, which was computed by FeatureCounts 2.0 at the genebody [300 base pair (bp) downstream of the TSS to the TES) to the promoter (100 bp upstream to 300 bp downstream of the TSS). Empirical cumulative distribution functions and boxplots for the log_2_PRR were generated in R. Tracks were visualized in igv 2.13.2 (Broad Institute).

#### RNA sequencing

Preprocessing was performed using CETO Toolbox (*45*). Sequence quality was assessed using FastQC v0.11.2 (*46*), and quality trimming was done using Trimmomatic (*47*) with parameters TRAILING:30 MINLEN:20. RNA-seq reads were aligned to the GRCh38 genome using STAR v.2.5.2 (*48*), and only uniquely mapped reads with a two-mismatch threshold were considered for downstream analysis and quantified to the gene level using HTSeq (*49*). Output BAM files were converted into bigWig track files to display coverage throughout the genome (in rpm). Gene count tables were constructed following Ensembl GRCh38.p13, release 98 (*42*). Raw read counts were normalized to remove technical differences and batch effects using R library RUVseq 1.26.0 (*50*), specifically the upper-quartile correction. Differential gene expression analysis was performed using DESeq2 1.32.0 (*49*). Genes with adjusted *P* values <0.05 (the Benjamini and Hochberg method) and an absolute log_2_FC ≥1 were considered differentially expressed.
