# Supplementary material for: Ectopic expression of testis-specific transcription elongation factor in driving cancer
Source: Sci Adv. 2025 Mar 14;11(11):eads4200. doi: 10.1126/sciadv.ads4200 (PMC11908497; doi:10.1126/sciadv.ads4200)
Supplement: Supplementary file 1 — Figs. S1 to S4 [file sciadv.ads4200_sm.pdf]

Science Advances
AAAS

Supplementary Materials for

Ectopic expression of testis-specific transcription elongation factor in driving cancer

Bin Zheng *et al.*

Corresponding author: Lu Wang, lu.wang1@northwestern.edu; Ali Shilatifard, ash@northwestern.edu

Sci. Adv. **11**, eads4200 (2025)
DOI: 10.1126/sciadv.ads4200

This PDF file includes:

Figs. S1 to S4

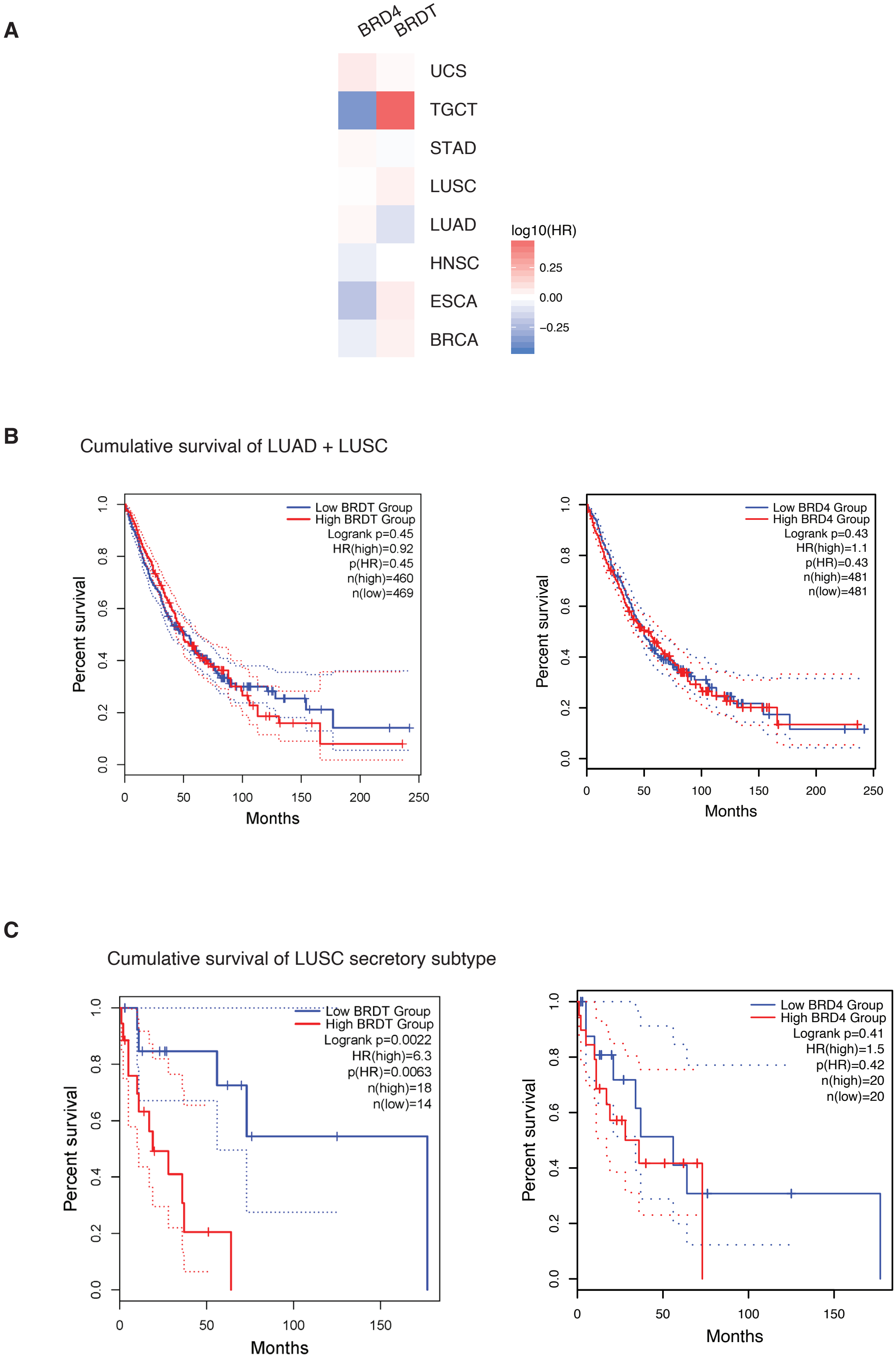
A
BRD4
BRDT
UCS
TGCT
STAD
LUSC
LUAD
HNSC
ESCA
BRCA
log10(HR)
0.25
0.00
−0.25
B
Cumulative survival of LUAD + LUSC
Low BRDT Group
High BRDT Group
Logrank p=0.45
HR(high)=0.92
p(HR)=0.45
n(high)=460
n(low)=469
Percent survival
Months
Low BRD4 Group
High BRD4 Group
Logrank p=0.43
HR(high)=1.1
p(HR)=0.43
n(high)=481
n(low)=481
Percent survival
Months
C
Cumulative survival of LUSC secretory subtype
Low BRDT Group
High BRDT Group
Logrank p=0.0022
HR(high)=6.3
p(HR)=0.0063
n(high)=18
n(low)=14
Percent survival
Months
Low BRD4 Group
High BRD4 Group
Logrank p=0.41
HR(high)=1.5
p(HR)=0.42
n(high)=20
n(low)=20
Percent survival
Months

Fig. S1. Analysis of patient data for BRD4 and BRDT in cancers

A. Heatmap of log10 normalized hazard ratios (HR) for patients with high expression of BRDT or BRD4 across different BRDT-expressing solid tumor types. HR were calculated as described in Fig. 1C.

B. Kaplan-Meier curves as described in Fig. 1C, for overall survival in all patients with LUAD or LUSC (including both defined and undefined subtypes).

C. Kaplan-Meier curves as described in Fig. 1C, for overall survival in patients with the defined secretory subtype of LUSC.

A

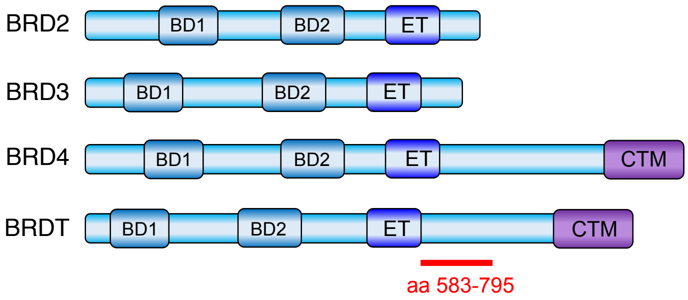

B

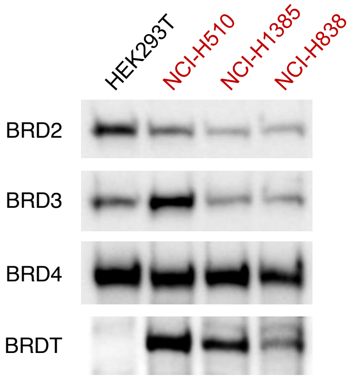

Pathway Enrichment Analysis

C

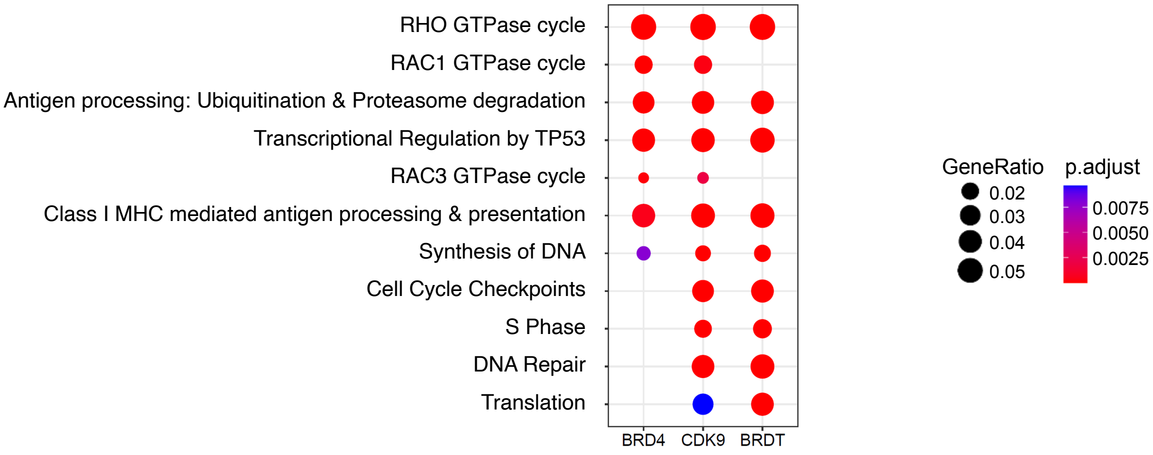

D

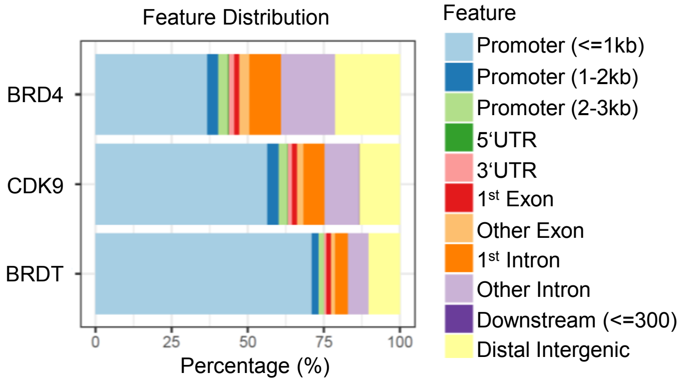

Fig. S2. Chromatin binding of BRD4 and BRDT

A. Schematic diagram illustrating the domain structure of BET (Bromodomain and Extra-Terminal domain) protein family members and the location of the peptide antigen (aa 583-795) used to generate the BRDT antibody, with position of bromodomains (BD1 and BD2) extra-terminal domain (ET) and the PTEFb-interacting C-terminal Motif (CTM, exclusive to BRD4 and BRDT) indicated.

B. Western blot for relative expression of BRD2, BRD3, and BRD4 proteins in HEK293T cells, the SCLC cell line NCI-H510, and the NSCLC cell lines NCI-H1385 (LUSC subtype) and NCI-H1838 (LUAD subtype), both of which were derived from female patients.

C. Pathway enrichment analysis of BRDT, BRD4, and CDK9 ChIP-seq signal peaks in NCI-H510 cells, indicating patterns of transcriptional regulation by BRDT and BRD4.

D. Genomic feature distribution analysis of BRDT, BRD4, and CDK9 ChIP-seq signal peaks in NCI-H510 cells, illustrating genome-wide distributions of chromatin-bound BRDT and BRD4.

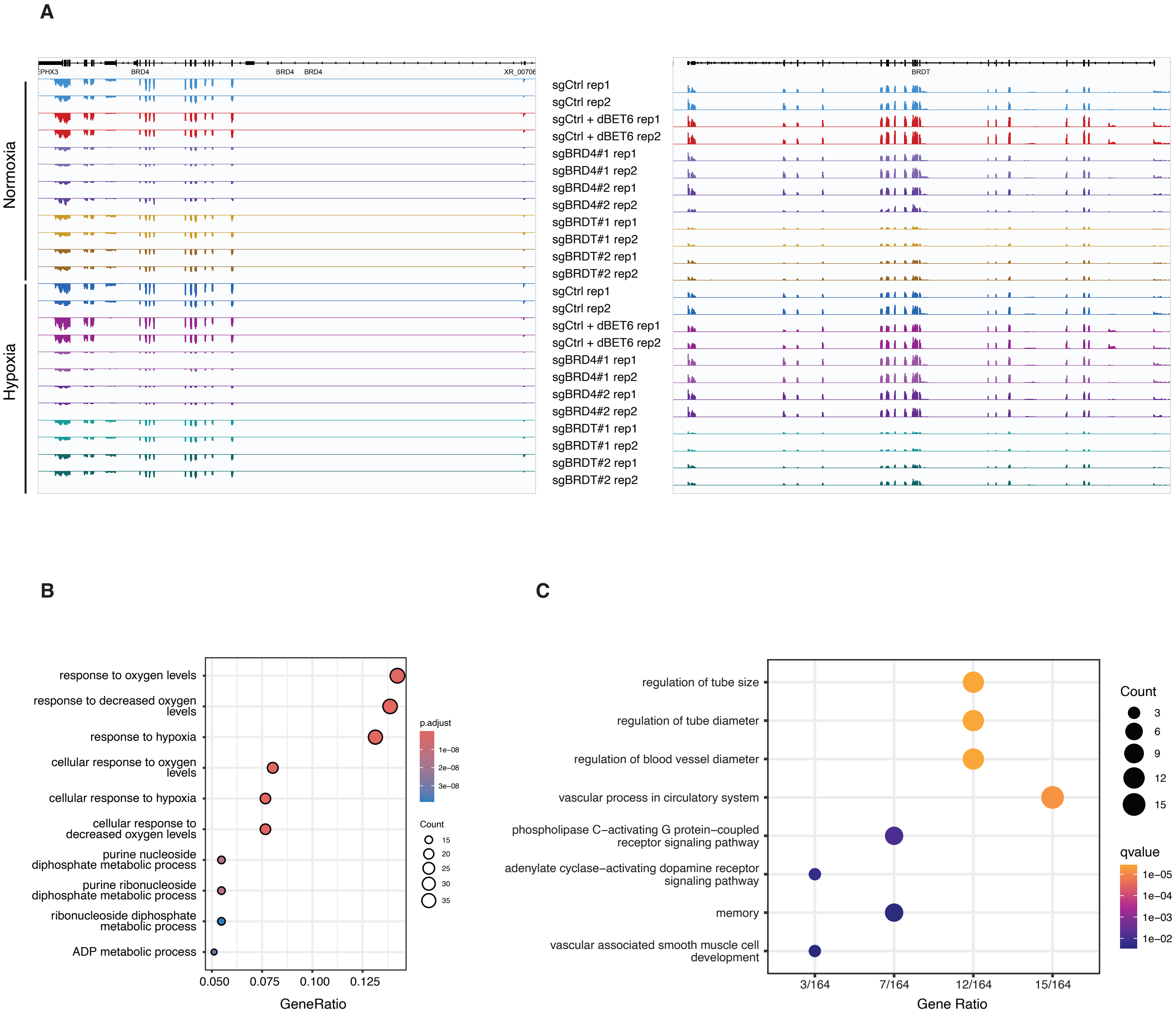
A
Normoxia
Hypoxia
EPHX3
BRD4
BRD4
BRD4
BRDT
sgCtrl rep1
sgCtrl rep2
sgCtrl + dBET6 rep1
sgCtrl + dBET6 rep2
sgBRD4#1 rep1
sgBRD4#1 rep2
sgBRD4#2 rep1
sgBRD4#2 rep2
sgBRDT#1 rep1
sgBRDT#1 rep2
sgBRDT#2 rep1
sgBRDT#2 rep2
sgCtrl rep1
sgCtrl rep2
sgCtrl + dBET6 rep1
sgCtrl + dBET6 rep2
sgBRD4#1 rep1
sgBRD4#1 rep2
sgBRD4#2 rep1
sgBRD4#2 rep2
sgBRDT#1 rep1
sgBRDT#1 rep2
sgBRDT#2 rep1
sgBRDT#2 rep2
B
response to oxygen levels
response to decreased oxygen levels
response to hypoxia
cellular response to oxygen levels
cellular response to hypoxia
cellular response to decreased oxygen levels
purine nucleoside diphosphate metabolic process
purine ribonucleoside diphosphate metabolic process
ribonucleoside diphosphate metabolic process
ADP metabolic process
0.050
0.075
0.100
0.125
GeneRatio
p.adjust
1e−08
2e−08
3e−08
Count
15
20
25
30
35
C
regulation of tube size
regulation of tube diameter
regulation of blood vessel diameter
vascular process in circulatory system
phospholipase C−activating G protein−coupled receptor signaling pathway
adenylate cyclase−activating dopamine receptor signaling pathway
memory
vascular associated smooth muscle cell development
3/164
7/164
12/164
15/164
Gene Ratio
Count
3
6
9
12
15
qvalue
1e−05
1e−04
1e−03
1e−02

Fig. S3. Interrogating roles of BRD4 and BRDT for transcriptional response to hypoxia

A. Track visualization of RNA-seq signal at the *BRD4* and *BRDT* loci for the two replicates of the hypoxia response study, confirming efficient knockdown of these genes by sgRNA.
B. GO terms enriched among genes that are significantly upregulated in response to hypoxia in untreated sgCtrl cells.
C. GO terms enriched among the genes for which transcriptional induction in response to hypoxia is redundantly mediated by both BRD4 and BRDT.

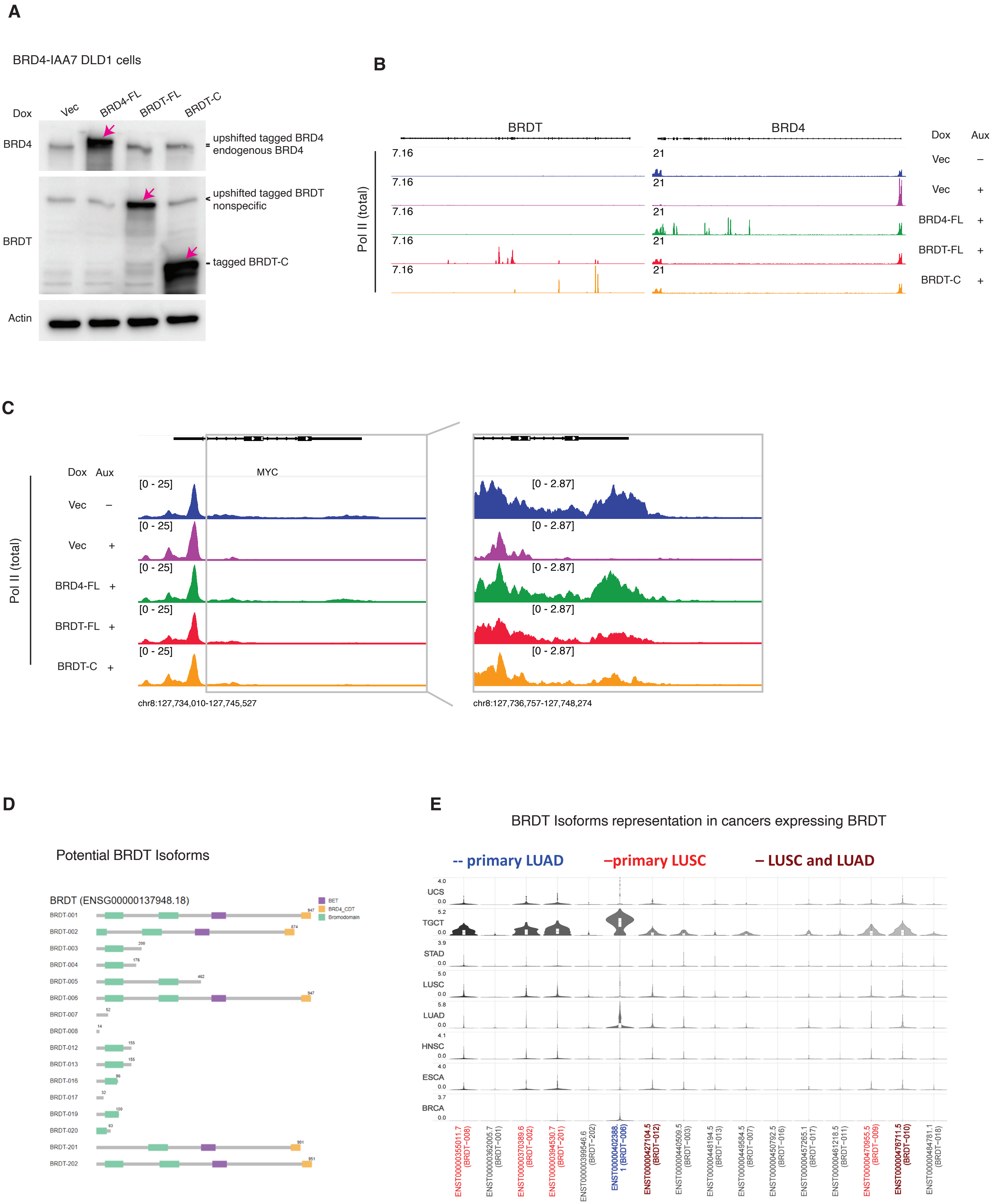

A
BRD4-IAA7 DLD1 cells
Dox
Vec
BRD4-FL
BRDT-FL
BRDT-C
BRD4
upshifted tagged BRD4
endogenous BRD4
upshifted tagged BRDT
nonspecific
BRDT
tagged BRDT-C
Actin
B
BRDT
BRD4
Pol II (total)
7.16
21
Dox
Aux
Vec
–
Vec
+
BRD4-FL
+
BRDT-FL
+
BRDT-C
+
C
Dox
Aux
MYC
Pol II (total)
Vec
–
Vec
+
BRD4-FL
+
BRDT-FL
+
BRDT-C
+
[0 - 25]
[0 - 2.87]
chr8:127,734,010-127,745,527
chr8:127,736,757-127,748,274
D
Potential BRDT Isoforms
BRDT (ENSG00000137948.18)
BET
BRD4_CDT
Bromodomain
BRDT-001
BRDT-002
BRDT-003
BRDT-004
BRDT-005
BRDT-006
BRDT-007
BRDT-008
BRDT-012
BRDT-013
BRDT-016
BRDT-017
BRDT-019
BRDT-020
BRDT-201
BRDT-202
947
874
200
176
462
947
52
14
155
155
96
32
100
63
901
951
E
BRDT Isoforms representation in cancers expressing BRDT
-- primary LUAD
–primary LUSC
– LUSC and LUAD
UCS
TGCT
STAD
LUSC
LUAD
HNSC
ESCA
BRCA
4.0
5.2
3.9
5.0
5.8
4.1
4.0
3.7
0.0
ENST00000355011.7 (BRDT−008)
ENST00000362005.7 (BRDT−001)
ENST00000370389.6 (BRDT−002)
ENST00000394530.7 (BRDT−201)
ENST00000399546.6 (BRDT−202)
ENST00000402388.1 (BRDT−006)
ENST00000427104.5 (BRDT−012)
ENST00000440509.5 (BRDT−003)
ENST00000448194.5 (BRDT−013)
ENST00000449584.5 (BRDT−007)
ENST00000450792.5 (BRDT−016)
ENST00000457265.1 (BRDT−017)
ENST00000461218.5 (BRDT−011)
ENST00000470955.5 (BRDT−009)
ENST00000476711.5 (BRDT−010)
ENST00000484781.1 (BRDT−018)

Fig. S4. BRDT releases promoter-proximal paused Pol II independently of its bromodomains

A. Western blot for the GFP-tagged BRD4-FL, BRDT-FL, and BRDT-C upon 2 days of Dox induction in BRDT-null BRD4-IAA7 DLD1 cells. GFP-tagged BRD4-FL upshifted to slightly above the endogenous one. GFP-tagged BRDT-FL upshifted to the same position as the main non-specific band for the home-made antibody.

B. Track visualization of Pol II ChIP-seq signal at the *BRDT* and *BRD4* gene loci for the conditions in Fig. 5C, illustrating successful expression of the indicated constructs.

C. Track visualization of Pol II ChIP-seq signal at the MYC locus (with enlarged view of signal at gene body) for the conditions in Fig. 5C.

D. Schematic diagram illustrating the putative domain structure of proteins resulting from alternative BRDT transcripts that are predicted to be protein-coding.

E. Expression levels of alternative BRDT transcripts (horizontal) in each of the cancer subtypes with BRDT expression in Fig. 1B (vertical). Isoforms primarily represented in LUAD versus LUSC are highlighted in blue, isoforms primarily represented in LUSC versus LUAD are highlighted in red, and isoforms similarly represented in both LUAD and LUSC are highlighted in maroon.
